# Diagnostic accuracy of diagnostic imaging for lumbar disc herniation in adults with low back pain or sciatica is unknown; a systematic review

**DOI:** 10.1186/s12998-018-0207-x

**Published:** 2018-08-21

**Authors:** Jung-Ha Kim, Rogier M. van Rijn, Maurits W. van Tulder, Bart W. Koes, Michiel R. de Boer, Abida Z. Ginai, Raymond W. G. J. Ostelo, Danielle A. M. W. van der Windt, Arianne P. Verhagen

**Affiliations:** 1 Department of General Practice, Erasmus University Medical Center, Rotterdam, The Netherlands; 2 Department of Family Medicine, Chung-ang University Medical Center, 102, Heukseok-ro, Dongjak-gu, Seoul, South Korea; 3 Department of Public Health, Erasmus University Medical Center Rotterdam, Rotterdam, The Netherlands; 4 Department of Health Sciences and EMGO-Institute for Health and Care Research, Faculty of Earth & Life Sciences, VU University Medical Centre, Amsterdam, The Netherlands; 5 Department of Epidemiology and Biostatistics and EMGO-Institute for Health and Care Research, VU University Medical Centre, Amsterdam, The Netherlands; 6 Department of Radiology, Erasmus University Medical Centre, Rotterdam, Netherlands; 7 Arthritis Research UK Primary Care Centre, Institute for Primary Care and Health Sciences, Keele University, Staffordshire, UK; 8 School of Physiotherapy, Graduate school of Health, University Technology Sydney, Sydney, Australia

**Keywords:** Diagnostic accuracy, Systematic review, Lumbar disc herniation, Diagnostic imaging, Low back pain

## Abstract

**Main text:**

We aim to summarize the available evidence on the diagnostic accuracy of imaging (index test) compared to surgery (reference test) for identifying lumbar disc herniation (LDH) in adult patients.

For this systematic review we searched MEDLINE, EMBASE and CINAHL (June 2017) for studies that assessed the diagnostic accuracy of imaging for LDH in adult patients with low back pain and surgery as the reference standard. Two review authors independently selected studies, extracted data and assessed risk of bias. We calculated summary estimates of sensitivity and specificity using bivariate analysis, generated linked ROC plots in case of direct comparison of diagnostic imaging tests and assessed the quality of evidence using the GRADE-approach.

We found 14 studies, all but one done before 1995, including 940 patients. Nine studies investigated Computed Tomography (CT), eight myelography and six Magnetic Resonance Imaging (MRI). The prior probability of LDH varied from 48.6 to 98.7%. The summary estimates for MRI and myelography were comparable with CT (sensitivity: 81.3% (95%CI 72.3–87.7%) and specificity: 77.1% (95%CI 61.9–87.5%)). The quality of evidence was moderate to very low.

**Conclusions:**

The diagnostic accuracy of CT, myelography and MRI of today is unknown, as we found no studies evaluating today’s more advanced imaging techniques. Concerning the older techniques we found moderate diagnostic accuracy for all CT, myelography and MRI, indicating a large proportion of false positives and negatives.

## Main text

### Introduction

Approximately 5–15% of patients with low back pain suffer from lumbar disc herniation (LDH) [1, 2]. LDH is the most common spine disorder requiring surgical intervention [3, 4]. Clinical guidelines recommend history taking and physical examination to rule out LDH diagnosis [4]. However, the diagnostic accuracy of both history taking and physical examination is still insufficient [5, 6]. Diagnostic imaging in patients with back pain and/or leg pain is often used to assess nerve root compression due to disc herniation or spinal stenosis and cauda equina syndrome [7–10]. Furthermore, diagnostic imaging can also be used to identify the affected disc level before surgery [11].

Diagnostic imaging can be done by Magnetic Resonance Imaging (MRI), Computed Tomography (CT), X-ray and myelography. Currently MRI is the imaging modality of choice, as it has the advantage of not using ionising radiation and has good visualizing capacities especially of soft tissue [9, 12]. CT is often used and available for detection of morphologic changes and has a well-recognized role in the diagnosis of herniated discs [13, 14]. Compared to MRI, CT is cheaper, the total testing time is shorter, and the availability of CT scanners is larger in hospital settings, but has the drawback of exposure to ionising radiation. Myelography involves injection of contrast medium in the lumbar spine, followed by X-ray, CT or MRI projections [15]. For certain conditions (e.g. metal implants or malalignment of the spine) myelography might replace MRI as the imaging modality of choice [16]. Plain radiography (X-ray) is the most commonly used technique due to its relative low cost and ready availability [9, 17–19].

However, the evidence for diagnostic accuracy of diagnostic imaging for LDH is still unclear [20, 21]. In addition, discordance between patients’ clinical findings and MRI findings is also reported [22, 23]. We have performed a large study evaluating the evidence om diagnostic accuracy of MRI and CT for all kinds of lumbar pathologies compared to various reference standards [12, 24]. The aim of the current review is to more specifically summarize and compare the evidence on the diagnostic accuracy of diagnostic imaging (CT, X-rays, myelography and MRI) identifying LDH in patients with low back pain and/or leg pain with surgery as a reference standard.

## Methods

### Design

A systematic review and meta-analysis, according to the guidelines of the Cochrane handbook of systematic reviews of diagnostic test accuracy studies [25]. The protocol was registered in PROSPERO (2015:CRD42015027687).

### Search strategy

We conducted the search in MEDLINE, EMBASE, and CINAHL (untill 1 June 2017) without language restriction (see Appendix 1). The search strategy was designed in collaboration with a medical information specialist. In addition, reference lists of relevant review articles as well as all retrieved relevant publications on diagnostic test accuracy studies were checked to identify any potentially missed articles.

### Study selection

We applied the following selection criteria: a) both prospective and retrospective cohort and case-control studies; b) adults with low back and/or leg pain with lumbar disc herniation as the suspected underlying pathology; c) Index tests were MRI, X-ray, myelography or CT; d) Reference standard was surgery; e) Data to generate 2 × 2 table; f) Published full reports, preferably in English, Dutch or German language.

We defined LDH as herniated nucleus pulposus, including protruded, extruded or sequestrated disc, causing nerve root compression. Two of the review authors (RvR/RO/BK/JHK/MB) independently selected first titles and abstracts and assessed relevant full papers. We used consensus to resolve disagreements; in cases of persisting disagreement a third review author (AV) was consulted.

### Risk of bias assessment

Pairs of review authors (MvT/BK/RvR/JHK) independently performed risk of bias assessment using the Quality Assessment of Diagnostic Accuracy Studies (QUADAS)-2 tool [26]. In the flow and timing domain, we considered a time period between index test and reference standard of 1 week or less appropriate. Risk of bias and concerns about applicability of each domain were classified as low, high or unclear risk. Consensus was reached by discussion of discrepancies between the two reviewers. If discrepancies persisted, we consulted a third reviewer (AV).

### Data extraction

Pairs of review authors (MvT/BK/JHK/RvR) independently performed data extraction using a standardised form. We extracted data on author, year of publication and journal; study design and setting; study population; pathology considered, age, gender, numbers of subjects for inclusion in study and analysis, patient selection, level of measurement (patient or disc). Also, we obtained data on index and reference test characteristics; including type of test, year; methods of execution, cut- off values, positivity thresholds and outcome scales; diagnostic parameters; diagnostic two-by-two table or parameters to reconstruct this table.

### Statistical analysis

For each included study we calculated sensitivity and specificity (and 95% confidence intervals (CI)) preferably on patient level data using the data from two-by-two tables. We conducted a meta-analysis separately for each of the index tests using a bivariate analysis. We chose the bivariate random-effects approach, because it incorporates both within and between study variation of sensitivity and specificity together with any correlation that might exist between sensitivity and specificity [27]. We present summary point estimates of sensitivity and specificity (and 95% confidence region) and the results were plotted in receiver operating characteristic (ROC) space [28]. When possible we generated linked ROC plots in case of pairs of diagnostic imaging tests, when both tests had been evaluated in the same study. Meta-regression was used to evaluate whether there is a difference in test accuracy between different imaging techniques or between patient level data and disc level data [29]. Analysis was carried out using STATA 13.1 software.

Two reviewers (JHK, AV) assessed the quality of the evidence for each index test using the Grading of Recommendations Assessment, Development, and Evaluation (GRADE) working group criteria [28, 30]. Disagreements were resolved by a third review author (MB/DvdW). The quality of evidence is categorized as high, moderate, low, or very low [31]. The quality of the evidence started at high and is reduced by one level for each of the following domains not met: limitations of the study design (> 25% of participants in studies with two or more domains with high risk of bias); indirectness (> 25% of participants in studies with serious applicability concerns); inconsistency (unexplained variation in sensitivities and specificities across the studies [32]); imprecision (wide confidence interval of the sensitivity and specificity in > 25% of the studies); and publication bias [33].

## Results

### Literature search

**Fig. 1:**
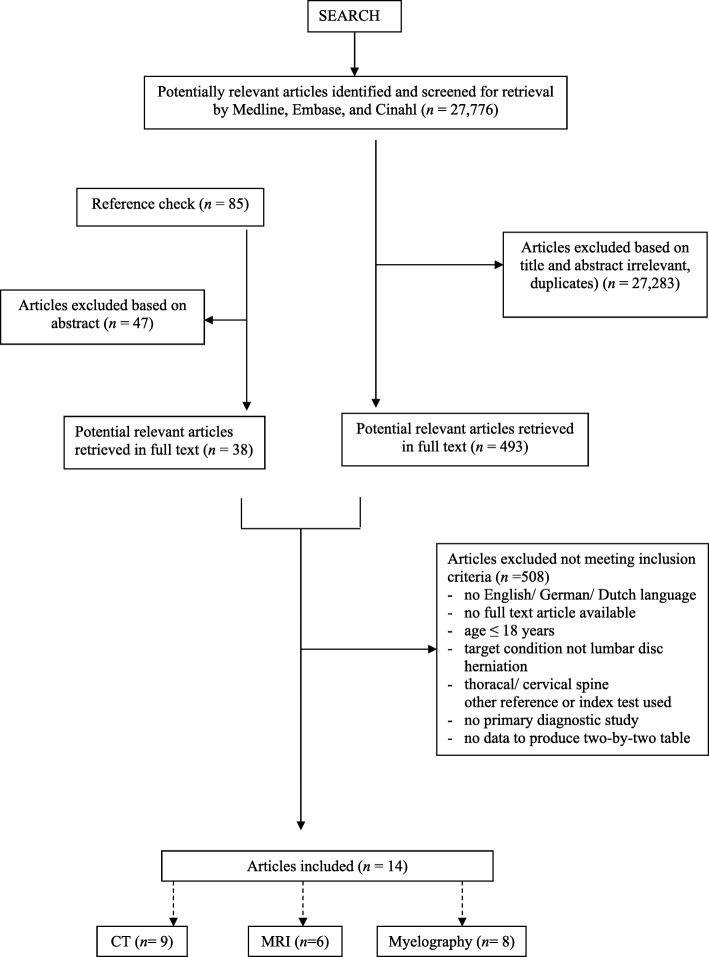
Flow chart of selected articles

A total of 27,776 citations were obtained. Finally, 14 studies met our selection criteria (Fig. 1). No studies were excluded based on the language. Of these, nine studies investigated CT [34–42], eight myelography [34, 37–39, 41, 43], six MRI [36, 39, 43–46], and none assessed X-ray. All studies were performed in secondary care settings, such as neurological clinics or pain clinics; three studies [41, 43, 47] were retrospective (Table 1). All but one study evaluated old imaging techniques as they were published between 1982 and 1994, one study evaluating MRI was published in 2006 [46].

**Table 1:** Study characteristics

Author	Design and setting	Patients	Target condition (prevalence)	Level of measurement	Index test	Note
Aejmelaus 1984 [47]	Retrospective, secondary care, Finland	200 patients; 54.5% male (of *n* = 200), mean age 43.8 (range 14–82)	Diagnosis of disc herniation (68.4%)	Patient level; 95 patients on lumbar spine surgery	Myelo: contrast medium metrizamide (Amipaque, 170-200 mg iodine/ml)	
Bernard 1994 [44]	Prospective study, secondary care, USA	33 patients persistent/ recurring symptoms after lumbar surgery: 61% male; age range: 23–74 years	Recurrent lumbar disc herniation (69.7%)	Patient level	MRI: 0.5Tesla MRI (29 patients) or 1.5Tesla MRI (4 patients) including spin echo T1 and T2 sagittal images	2 observers assessed each patient = 66 responses
Birney 1992 [45]	Prospective study, secondary care, USA	90 patients with LBP or radicular pain refractory to ≥3 months of conservative, non operative treatment; 57 underwent surgery; 53% males age range 20–71 years	Lumbar disc herniation and/ or degenerative disc disease (98.7%)	Disc level; 76 disc levels of 57 operated patients	MRI: 0.35Tesla MRI. Axial images and sagittal images	
Bischoff 1993 [43]	Retrospective study, secondary care, USA	57 patients for lumbar spine surgery; 51% male; age range: 20–79 years	HNP (48.6%)	Disc level; 72 levels assessed of 47 operated patients	Myelo: infusion of 16 ml Omnipaque 180 solution MRI: 1.5Tesla MRI; sagittal and axial T1 and T2 weighted images	
Chawalparit 2006 [46]	Prospective study, secondary care, Thailand	123 LBP patients and suspected lumbar disc herniation; 50% male; age range: 21–60 years	Lumbar disc herniation (69.7%)	Patient level; 33 operated patients	MRI: full protocol; 1.5Tesla; sagittal T1 weighted images, sagittal T2 weighted images and axial T2 weighted images	54 patients treated conservatively and 36 lost to follow up; excluded from analysis
Claussen 1982 [34]	Prospective, Secondary care, Germany	77 patients with suspected disc prolapse, 46.7% male;	Disc prolapse (92.3%)	Patient level; 26 patients operated	CT: Somatom II, 10s; 125 kV, 460mHz	
					Myelo: metrizamide (amipaque)	
Firooznia 1984 [35]	Prospective, Secondary care, Germany	100 patients who underwent surgery for sciatica: 61% male, mean age 49 (19–76) years	Disc prolapse (90.5%)	Disc level; 116 levels assessed of 100 patients	CT: GE 8800 CT/T, 25 cm circular calibration, 250-400 mA, 120 kVp, 9.6 s	
Forristall 1988 [36]	Prospective, Secondary care, USA	32 patients with suspected lumbar disc herniation: 78% male, mean age 45 (22–74) years	HNP with neural compression (77.4%)	Disc level; 31 levels assessed in 25 operated patients	CT: Picker 1200 Synerview, 14 cm, 65 mA, 130 kV, 5 mm slice thickness, 5 ml of Amipaque 180 mg/ml	
					MRI: 1.5Tesla MRI sagittal T1 and T2 weighted images; Proton density and T2 weighted axial images	
Gillstrom 1986 [37]	Prospective, Secondary care, Sweden	90 patients with suspected herniated discs 59.4% male, age range 23–74 years	Lumbar disc herniation	Patient level; 37 operated patients	CT: General Electris GT/T 8800 unit	
					Myelo: Metrizamide contrast solution	
Jackson 1989 I [38]	Prospective, Secondary care, USA	124 patients with LBP and leg pain, refractory to conservative management: 70% male, mean age 43 (21–76) years	HNP: protruded, extruded, and sequestrated disc (54.1%)	Disc level; 231 levels assessed of 124 patients	CT: Siemens Somatom, 5 mm slice thickness with 1 mm overlap	
					Myelo: infusion of 14 ml metrizamide (Amipaque) of 180 mg iodine/ml	
Jackson 1989 II [39]	Prospective, Secondary care, USA	59 patients with LBP and leg pain refractory to conservative management: 56% male, mean age 40 (18–70) years	HNP: protruded, extruded, and sequestrated disc (49.2%)	Disc level; 120 levels assessed of 59 patients	CT: Siemens Somatom, 5 mm slice thickness with 1 mm overlap using bone and soft tissue settings	
					Myelo: infusion of 14 ml iohexol (Omnipaque) of 180 mg iodine/ml	
					MRI: 1.5Tesla MRI Sagittal T1 and T2 weighted images and axial T1 weighted images	
Milano 1991 [40]	Prospective, Italy	40 surgical patients; 57.5% male; mean age 43 (range 27–60)	Lumbar intervetrebral disc disease (50%)	Disc level; 80 discs examined	CT: Somaton DR CT scan, slices of 4 mm	
Schaub 1989 [41]	Retrospective, Secondary care, Swiss	29 patients with recurring symptoms after lumbar disk surgery: 48% male, mean age 49 (SD:13) years	HNP (62.1%)	Patient level	CT: No information	
					Myelo: No information	
Schipper 1987 [42]	Prospective, Secondary care, Netherlands	235 patients with radiating leg pain, referred to the neurosurgical department: 61% male, mean age 43 years	Lumbar disc herniation: (83.8%)	Patient level	CT: Philips Tomoscan 350, 200 As, 120 kV, 3 mm slice thickness	
					Myelo: 15 ml Iopamiro 200LBP: low back pain	

*HNP* Hernia nucleus pulposis

### Population

A total of 940 patients receiving surgery were included. Overall 1289 patients were involved in these studies but the reference standard was not performed in 349 patients. The patients (14 to 82 years) all had clinical findings consistent with LDH. Seven studies (*n* = 288) [34, 37, 41, 42, 44, 46, 47] were analyzed on patient level; others analyzed disc levels (Table 1).

### Risk of bias

**Fig. 2:**
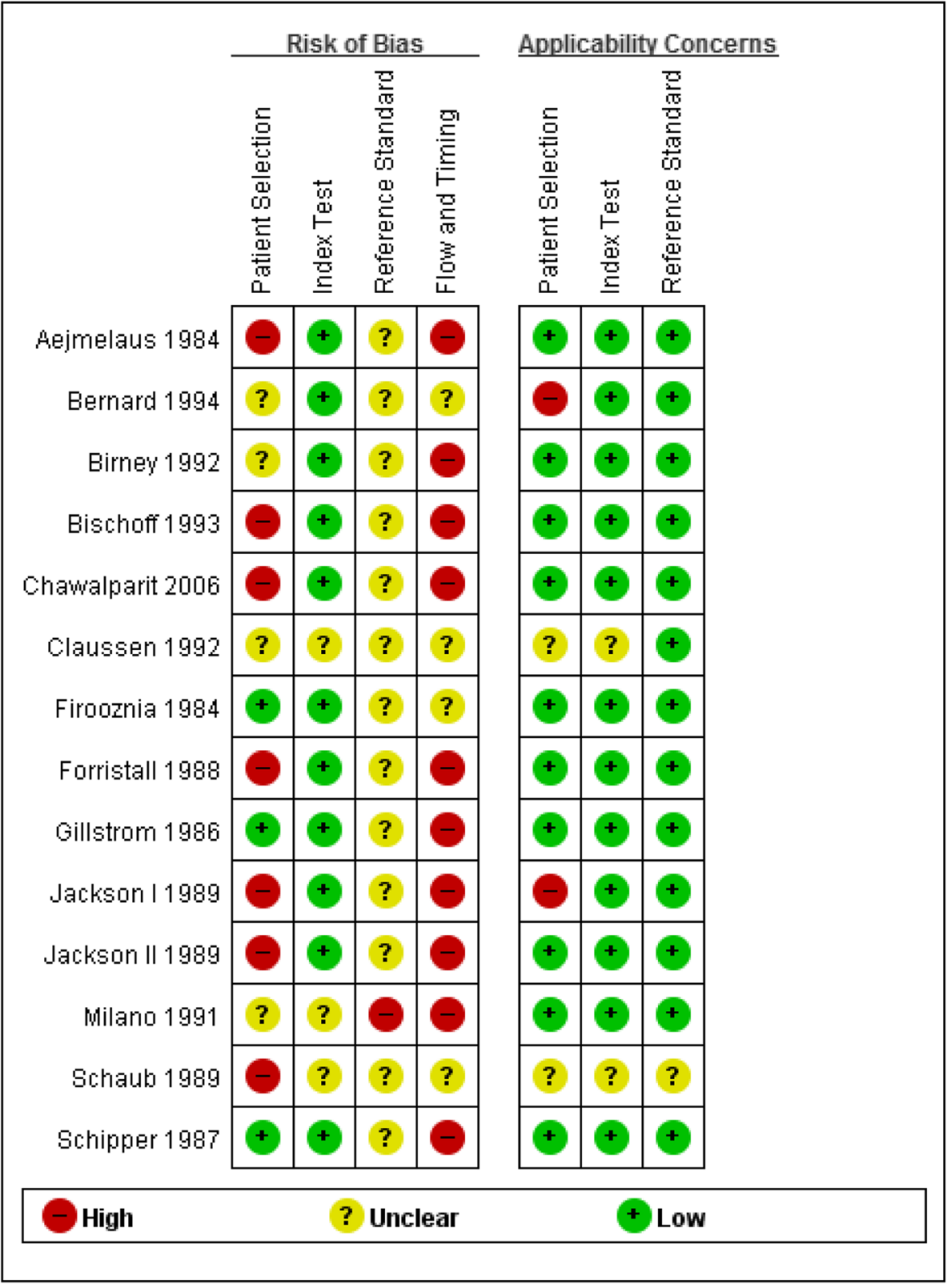
Assessment of risk of bias for each included study

Although we only selected studies using surgery as a reference standard, none of the studies were assessed as having low risk of bias (RoB) related to the reference standard, mainly because it was unclear whether results of the reference standard had been interpreted without knowledge of imaging results (Fig. 2). Seven studies were considered to have high RoB related to patient selection, as patients had not clearly been selected using consecutive or random sampling. Only two studies reported a time-interval between index test and reference standard, which were 3 months and 9 months, respectively [44, 47].

### Diagnostic accuracy

#### Computed tomography

**Fig. 3:**
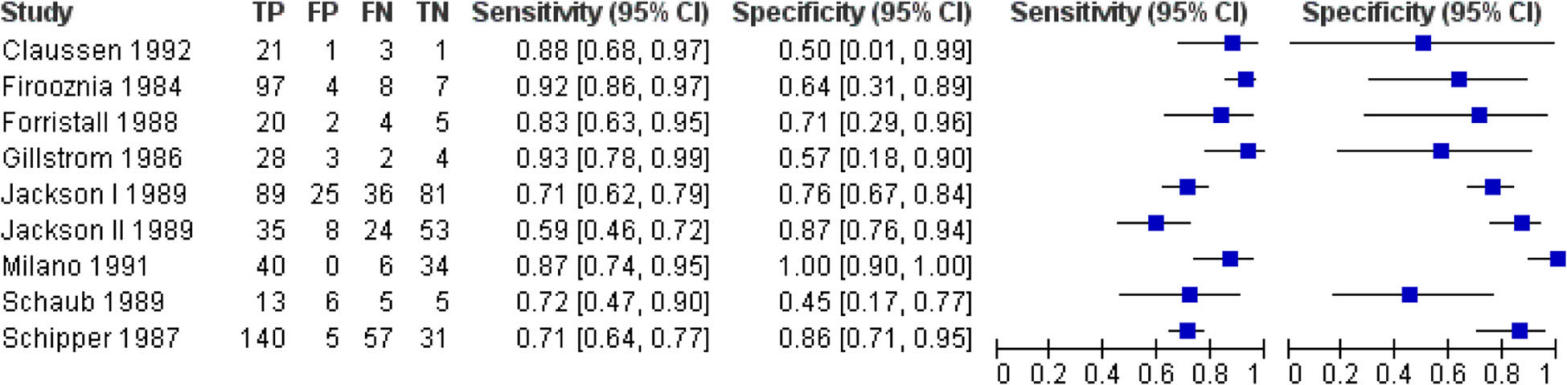
Forest plot of the diagnostic accuracy of CT in the identification of lumbar disc herniation

**Fig. 4:**
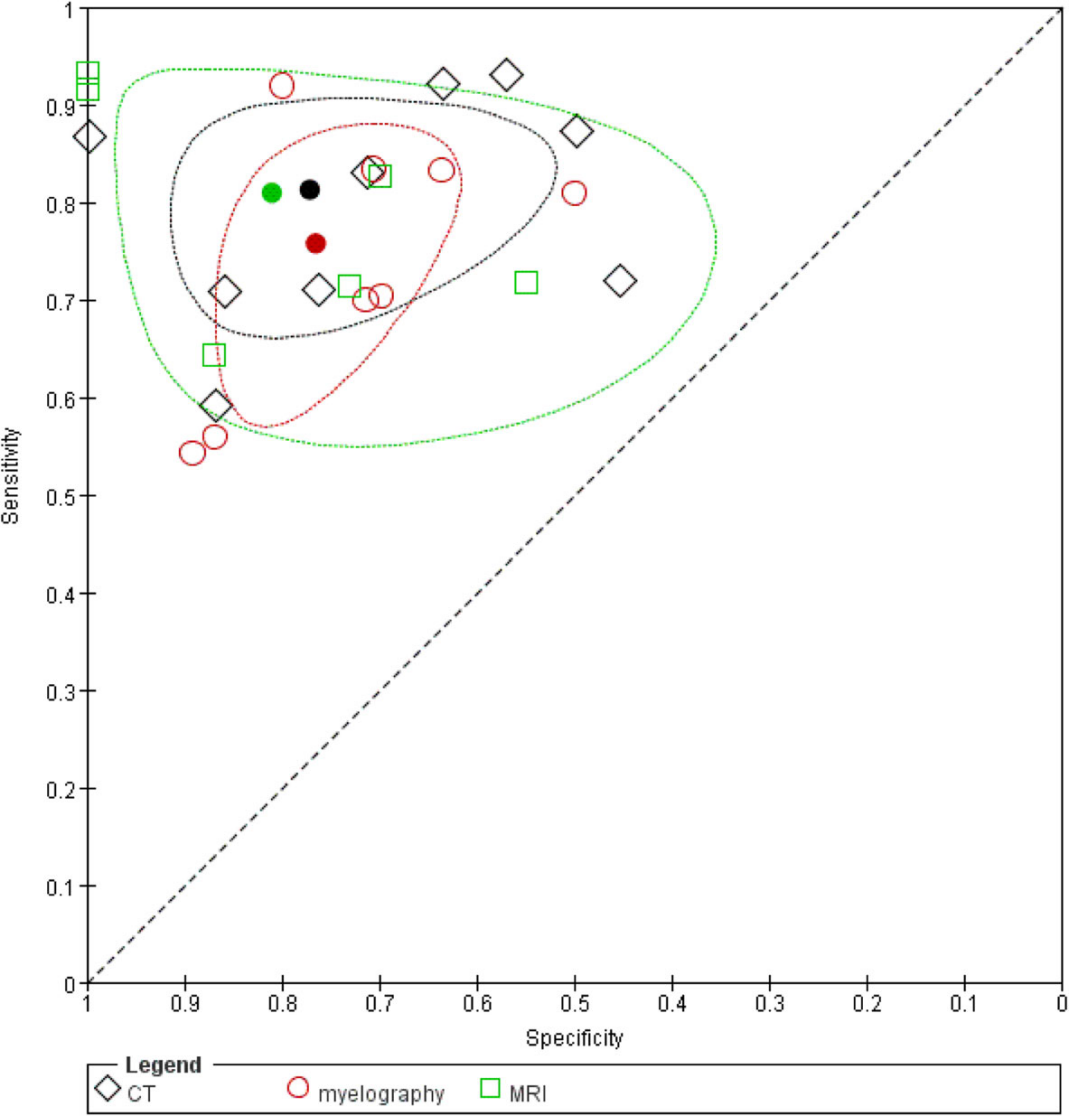
Summary ROC plots of sensitivity and specificity of all studies

Nine studies, with four studies with measurements on patient level (327 patients) [34, 37, 41, 42] and a total of 578 discs explorations [35, 36, 38–40], were included. The mean prior probability of LDH was 72.0% (range 49.2–92.3%). The sensitivity and specificity ranged from 59 to 93% and from 45 to 100%, respectively (Fig. 3). The summary estimates were 81.3% (95%CI: 72.3–87.7%) for sensitivity and 77.1% (95%CI: 61.9–87.5%) for specificity (Fig. 4). We found no inconsistency as an inverse correlation between logit-transformed sensitivity and logit-transformed specificity was shown (estimate = − 0.2649). There were no differences in summary estimates for sensitivity and/or for specificity between patient level data and disc level data (chi-square = 2.52, 2df, *P* = 0.28).

We found a moderate quality evidence (downgraded because of limitations in study design) for the accuracy of CT (Table 2).

**Table 2:** GRADE evidence for diagnostic accuracy of lumbar disc herniation

	Study design	Indirectness	Inconsistency	Imprecision	Publication bias	Quality
CT						
9 studies	Serious limitation^a^	No^b^	No^c^	No^d^	No^e^	Moderate
Myelography						
8 studies	Serious limitation^a^	No^b^	No^c^	No^d^	No^e^	Moderate
MRI						
6 studies	Serious limitation^a^	No^b^	Serious limitation^c^	Serious limitation^d^	No^e^	Very low

^a^More than 25% of participants in studies with two or more high risk of domains among four risk of bias domains

^b^Studies done in a hospital setting. It was not considered as a serious applicability concern because only surgery was a reference standard

^c^It was evaluated by a correlation between logit-transformed sensitivity and logit-transformed specificity. ^d^Wide confidence interval of the sensitivity and specificity in more than 25% of the studies

^e^The possibility of publication bias is not excluded but it was not considered sufficient to downgrade the quality of evidence

#### Myelography

**Fig. 5:**
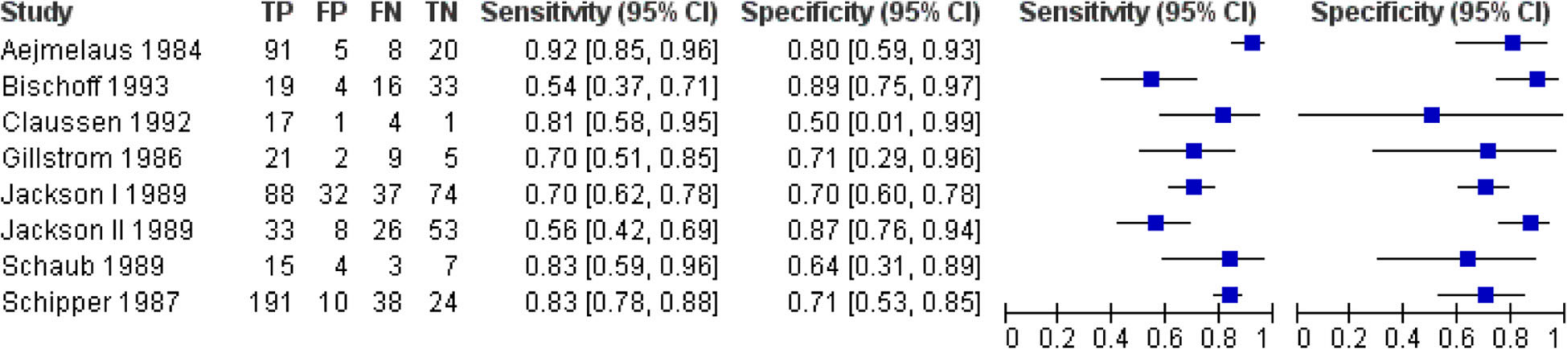
Forest plot of the diagnostic accuracy of myelography

Eight studies, with five studies with measurements on patient level (422 patients) [34, 37, 41, 42, 47] and a total 423 disc explorations [38, 39, 43], were included. The mean prior probability of LDH was 69.2% (range: 49.2–91.3%). The sensitivity and specificity ranged from 54 to 92% and from 50 to 89%, respectively (Fig. 5). We found a summary estimate of 75.7% (95%CI: 64.9–84.1%) for sensitivity and 76.5% (95%CI: 67.8–83.4%) for specificity (Fig. 4). We found no inconsistency (estimate = − 0.7644). There was a difference in summary estimate for sensitivity between patient level data (83.9% (95%CI: 76.4–89.3%)) and disc level data (61.1% (95%CI: 50.2–71.0%)) (chi-square = 9.23, 1df, *P* = 0.002), but not for specificity (chi-square = 1.26, 1df, *P* = 0.26).

We conclude that there is moderate quality evidence for the accuracy of myelography (downgraded because of limitations in study design) (Table 2).

#### Magnetic resonance imaging

**Fig. 6:**
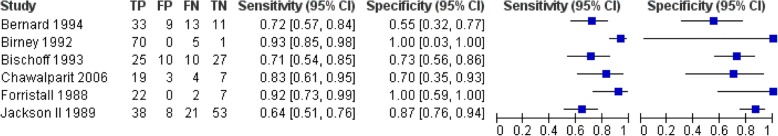
Forest plot of the diagnostic accuracy of MRI

Six studies, with two studies with measurements on patient level (66 patients) [44, 46] and a total 299 disc explorations [36, 39, 43, 45], were included. In these studies the mean prior probability of LDH was 68.9% (range: 48.6–98.7%). The sensitivity and specificity ranged from 64 to 93% and from 55 to 100%, respectively with wide confidence intervals (imprecision) (Fig. 6). The summary estimate was 80.9% (95%CI: 68.8–89.1%) for sensitivity and 81% (95%CI: 59.2–92.6%) for specificity (Fig. 4). Because of a positive correlation between logit-transformed sensitivity and logit-transformed specificity (estimate = 0.5516) we decided that there was inconsistency. It was not possible to examine a difference between patient level data and disc level data in sensitivity and specificity.

We conclude that there is very low quality evidence for the accuracy of MRI (downgraded by study design, inconsistency and imprecision) (Table 2).

### Comparing imaging techniques

#### CT versus Myelography

**Fig. 7:**
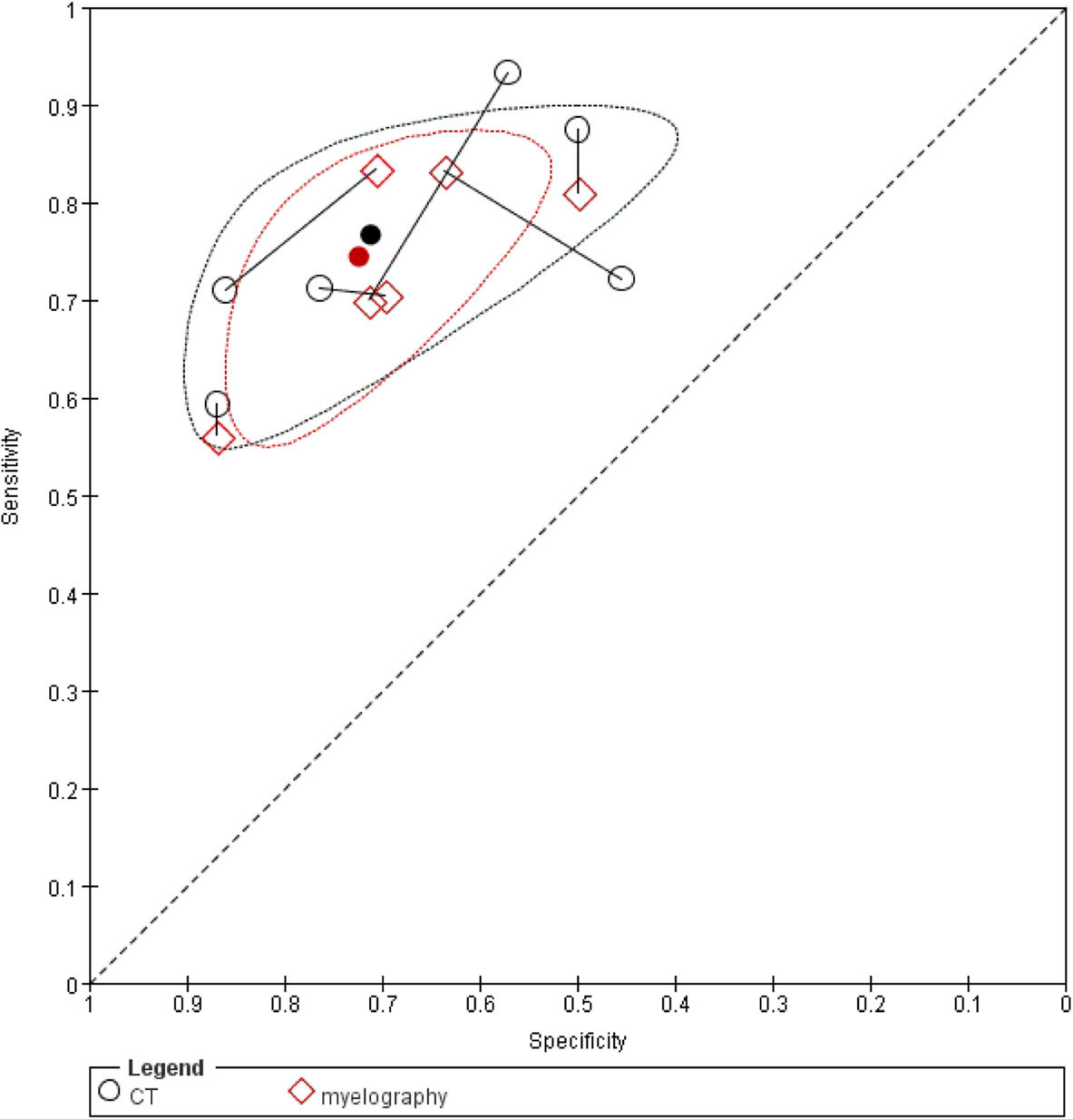
Summary ROC plots of CT versus myelography

Six studies evaluated CT and myelography (followed by plain radiography) in the same patient population and the linked results are plotted in ROC space (Fig. 7) [34, 37–39, 41, 42]. The summary estimate of sensitivity was 76.7% (95%CI: 66–84.8%) for CT and 74.4% (95%CI: 64.8–82.2%) for myelography. The summary estimate of specificity was 71.2% (95%CI: 55.2–83.2%) for CT and was 72.4% (95%CI: 62.5–80.4%) for myelography. These summary estimates were slightly lower compared to the ones based on all CT and myelography studies. We concluded that there is comparable accuracy for CT and myelography (chi square = 0.27, 2df, *P* = 0.87).

#### CT versus MRI

**Fig. 8:**
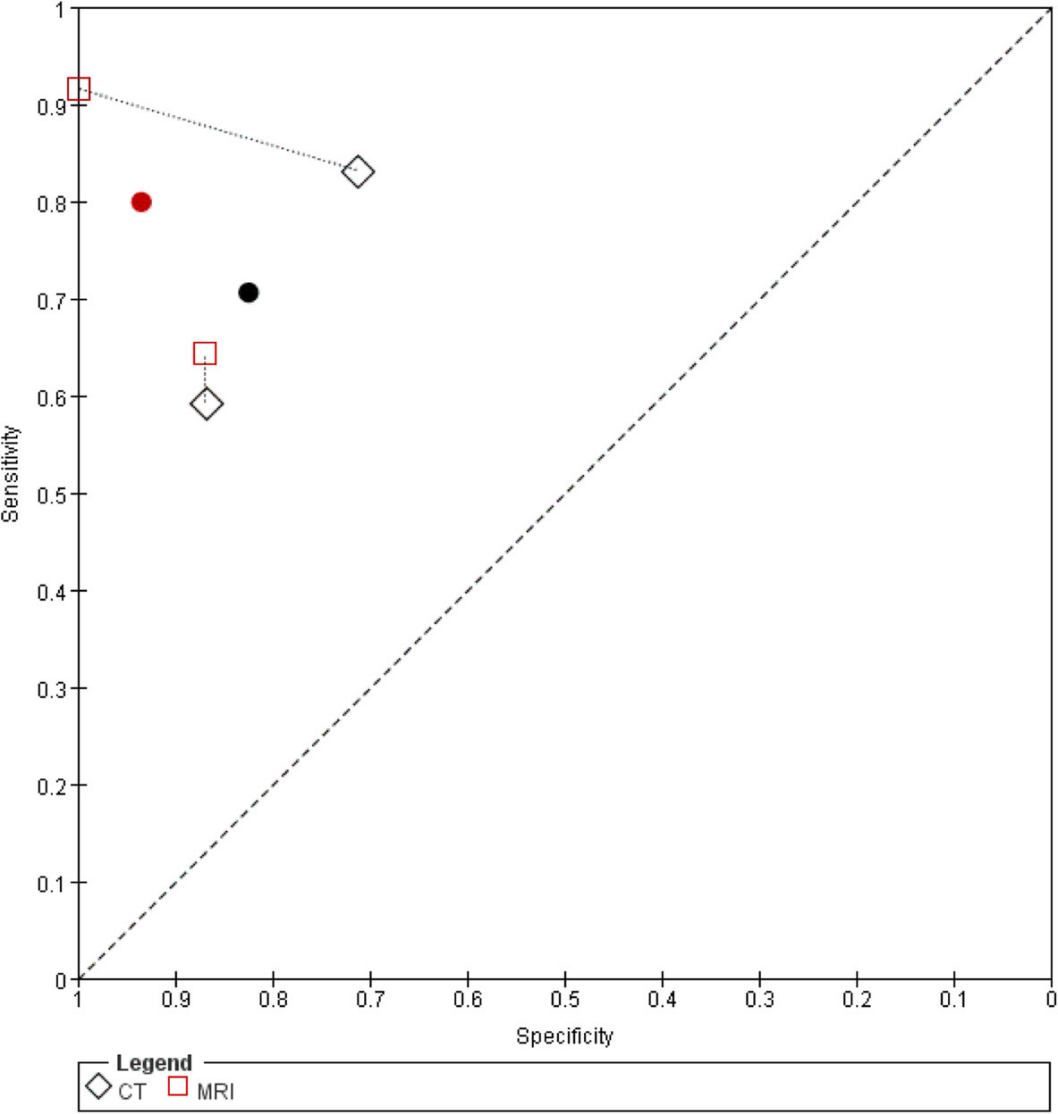
Summary ROC plots of CT versus MRI

Two studies evaluated CT and MRI (Fig. 8) [36, 39]. The summary estimate of sensitivity was 70.6% (95%CI: 49.5–85.5%) for CT and 80.0% (95%CI: 50.6–93.9%) for MRI. The summary estimate of specificity was 82.5% (95%CI: 63.3–92.7%) for CT and 93.5% (95%CI: 57.0–99.4%) for MRI. The results showed a comparable accuracy for CT and MRI (chi-square = 0.51, 2df, *P* = 0.78).

#### Myelography versus MRI

**Fig. 9:**
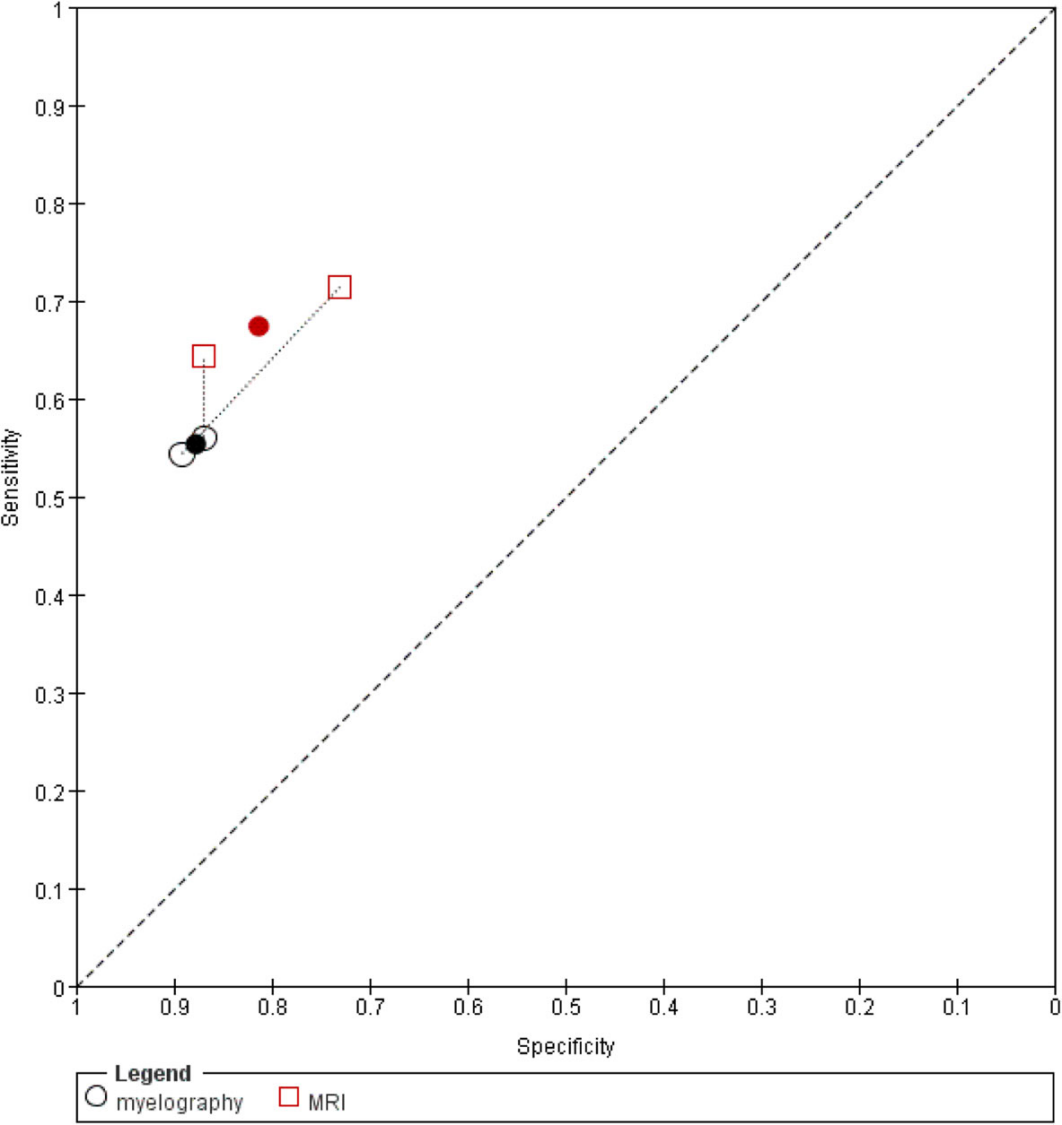
Summary ROC plots of myelography versus MRI

Two studies evaluated myelography and MRI (Fig. 9) [39, 43]. The summary estimate of sensitivity was 55.3% (95%CI: 45.2–65.0%) for myelography and 67.4% (95%CI: 56.6–76.7%) for MRI. The summary estimate of specificity was 87.8% (95%CI: 79.7–92.9%) for myelography and 81.3% (95%CI: 69.4–89.3%) for MRI. These results indicate comparable accuracy for myelography and MRI (chi-square = 3.59, 2df, *P* = 0.17).

## Discussion

We found 14 diagnostic accuracy studies including 940 patients and all evaluating rather old imaging techniques. Summary estimates of sensitivity and specificity of the different imaging techniques varied between 76 and 81%, with moderate to very low quality evidence. Furthermore, CT, myelography and MRI show comparable accuracy.

We found very low quality evidence for diagnostic accuracy of MRI. Even though MRI is more expensive, clinicians generally prefer MRI to CT, as it does not carry the risks associated with ionising radiation and unlike myelography, MRI is non-invasive [48]. MRI may also be more useful when surgical treatment is considered as it can identify tissue properties as well as anatomical structures [48]. These are most likely the reasons for suggesting MRI as the most appropriate test to confirm the presence of LDH in a recent guideline regardless its disappointing diagnostic accuracy.

### Strengths and weaknesses

Heterogeneity arises from several reasons. First, imaging techniques used in studies included old ones like 0.5Tesla [44] or 0.35Tesla MRI [45]. In clinical practice the results of diagnostic imaging are interpreted with knowledge of history items and physical examination. Furthermore, clinicians frequently state that imaging does not play a crucial role in predicting prognosis or deciding on a management strategy among patients with LDH [4]. This might be one of the reasons why there are no recent studies on the diagnostic accuracy of imaging techniques for detecting LDH. However, older techniques will probably identify less underlying causes of back pain than newer imaging techniques. Evaluation of diagnostic accuracy of advanced diagnostic equipment is therefore needed. Second, the included studies focussed on LDH, but classification of this pathology differed between studies [49]. For example, some studies defined LDH as protruded, extruded, and sequestrates disc [38, 39], but other studies were defined LHD as the presence of neuronal compression [35, 36, 42, 46]. There were some studies without a definition of LHD [37, 40]. Third, we combined disc level data with patient level data. Results at disc level including more than one disc level in the same patient may lead to smaller confidence intervals and possibly to an overestimation of diagnostic accuracy. Unexpectedly, confidence intervals were often wider in disc level data compared to patient level data. Fourth, the diagnostic accuracy in this study was possibly overestimated by a high prior probability (48.6 to 98.5%) of LDH. It was reported that about 4% of patients who present with low back pain in a primary care setting have a disc herniation [8]. The high prior probability results in selection bias. Furthermore, patient selection was unclear in many studies. This is important since the interpretation of the test result (posterior probability) depends on its sensitivity and specificity as well as the probability of the disease [50]. Lastly, the use of surgery as a reference standard can easily bias the results due to partial verification [51]. Surgery is often regarded as the best available reference standard. Not everyone is subjected to surgery but only those patients with a very strong suspicion based on clinical symptoms combined with the results of the diagnostic imaging of LDH which leads to (partial) verification bias. In this review, among 669 patients with suspected LDH, 349 (52.2%) patients did not undergo surgical treatment in seven studies [34, 36, 37, 43, 45–47]. Verification bias can lead to an increased diagnostic accuracy of the index test; i.e. it will show an increased sensitivity.

As far as we know, this is the first meta-analysis comparing diagnostic accuracy between different techniques in low back and/or leg pain with LDH as the suspected underlying pathology.

### Implications

Concerning practice we conclude that the diagnostic accuracy of today’s imaging techniques in unknown. This severely hampers the choice of techniques as well as the interpretation of the outcomes as no information is present concerning false positives or negatives. Future research should focus on the diagnostic accuracy of frequently used imaging techniques (diagnostic test accuracy studies) and on the place of diagnostic imaging within the clinical pathway (diagnostic modelling).

## Conclusion

In conclusion, we found no studies evaluating modern diagnostic imaging techniques. For the older techniques we found moderate quality evidence for moderate diagnostic accuracy of CT and myelography, and very low quality evidence for moderate diagnostic accuracy of MRI in patients with suspected lumbar disc herniation. The accuracy of CT, MRI and myelography is comparable.

## Appendix 1

### Search strategy DTA imaging in low back pain

**Embase.com**

(‘nuclear magnetic resonance imaging’/exp OR ‘computer assisted tomography’/exp OR radiography/exp OR ‘diagnostic imaging’/exp OR radiodiagnosis/de OR ((magnet* NEAR/3 resonance ) OR mri OR nmri OR ((mr OR nmr) NEAR/3 imag*) OR (comput* NEAR/3 tomograph*) OR ct OR cat OR radiogra* OR (x NEXT/1 ray*) OR ‘plain film’ OR myelogra* OR (diagnos* NEAR/3 imag*) OR radiodiagnos*):ab,ti) AND (backache/exp OR sciatica/exp OR (‘radicular pain’/exp AND (back/exp)) OR (((back OR sciatic* OR lowback OR lumb* OR sacroiliac*) NEAR/6 (ache OR pain* OR aching OR complaint* OR dysfunction* OR disabilit* OR trauma* OR symptom* OR injur* OR patholog* OR problem*)) OR (fail* NEAR/3 back NEAR/3 surg* ) OR backache* OR backpain* OR schiatica OR ischia* OR lumbago OR lumboischialgia OR ((radicular OR radiculalgi*) NEAR/6 (back OR spine* OR spinal*)) OR dorsalgi*):ab,ti) AND (‘spine disease’/exp OR ‘neurologic disease’/exp OR Osteoporosis/exp OR Osteoarthritis/exp OR Osteosclerosis/exp OR (((spin* OR vertebra* OR intervertebr* OR disc* OR disk* OR neurologic* OR nerve*) NEAR/3 (disease* OR injur* OR tumor* OR tumour* OR neoplas* OR cancer* OR malign* OR fracture* OR hernia* OR displace* OR protru* OR avuls* OR degenerat* OR Stenos* OR Osteophytos* OR entrap* OR compress* OR inflammat* OR disorder* OR rupture* OR disrupt*)) OR Radiculopath* OR polyradiculopath* OR Spondylarthrit* OR Spondyloarthrit* OR Spondylit* OR Spondylolisthes* OR Spondylolys* OR Discitis OR Osteoporo* OR Osteoarthrit* OR Osteosclero* OR Ankylos*):ab,ti) AND (‘cohort analysis’/exp OR ‘follow up’/exp OR ‘longitudinal study’/exp OR ‘prospective study’/exp OR ‘retrospective study’/exp OR ‘case control study’/exp OR ‘cross-sectional study’/de OR epidemiology/de OR (cohort* OR (follow* NEXT/1 up*) OR followup* OR longitudinal* OR prospectiv* OR retrospectiv* OR (case NEXT/1 control*) OR historical* OR epidemiolog* OR (cross NEXT/1 section*)):ab,ti) NOT ((juvenile/exp NOT adult/exp)) NOT ([animals]/lim NOT [humans]/lim) NOT ([Conference Abstract]/lim OR [Letter]/lim OR [Note]/lim OR [Editorial]/lim OR ‘systematic review’/exp OR ‘case report’/exp OR (‘systematic review’ OR ‘case report’):ti)

**Medline ovid**

(exp “magnetic resonance imaging”/ OR exp “Nuclear Magnetic Resonance”/ OR exp “Tomography, X-Ray Computed”/ OR exp radiography/ OR radiography.xs. OR “diagnostic imaging”/ OR radiodiagnosis/ OR ((magnet* ADJ3 resonance) OR mri OR nmri OR ((mr OR nmr) ADJ3 imag*) OR (comput* ADJ3 tomograph*) OR ct OR cat OR radiogra* OR (x ADJ ray*) OR “plain film” OR myelogra* OR (diagnos* ADJ3 imag*) OR radiodiagnos*).ab,ti.) AND (exp “back pain”/ OR sciatica/ OR (“Radiculopathy”/ AND (back/)) OR (((back OR sciatic* OR lowback OR lumb* OR sacroiliac*) ADJ6 (ache OR pain* OR aching OR complaint* OR dysfunction* OR disabilit* OR trauma* OR symptom* OR injur* OR patholog* OR problem*)) OR (fail* ADJ3 back ADJ3 surg* ) OR backache* OR backpain* OR schiatica OR ischia* OR lumbago OR lumboischialgia OR ((radicular OR radiculalgi*) ADJ6 (back OR spine* OR spinal*)) OR dorsalgi*).ab,ti.) AND (exp “Spinal Diseases”/ OR exp “Nervous System Diseases”/ OR Osteoporosis/ OR Osteoarthritis/ OR Osteosclerosis/ OR (((spin* OR vertebra* OR intervertebr* OR disc* OR disk* OR neurologic* OR nerve*) ADJ3 (disease* OR injur* OR tumor* OR tumour* OR neoplas* OR cancer* OR malign* OR fracture* OR hernia* OR displace* OR protru* OR avuls* OR degenerat* OR Stenos* OR Osteophytos* OR entrap* OR compress* OR inflammat* OR disorder* OR rupture* OR disrupt*)) OR Radiculopath* OR polyradiculopath* OR Spondylarthrit* OR Spondyloarthrit* OR Spondylit* OR Spondylolisthes* OR Spondylolys* OR Discitis OR Osteoporo* OR Osteoarthrit* OR Osteosclero* OR Ankylos*).ab,ti.) AND (“Epidemiologic Studies”/ OR exp “Cohort Studies”/ OR “Case-Control Studies”/ OR “cross-sectional studies”/ OR (cohort* OR (follow* ADJ up*) OR followup* OR longitudinal* OR prospectiv* OR retrospectiv* OR (case ADJ control*) OR historical* OR epidemiolog* OR (cross ADJ section*)).ab,ti.) NOT ((exp child/ NOT exp adult/)) NOT (exp animals/ NOT humans/) NOT ((Congresses OR Letter OR Notes OR Editorials).pt. OR “systematic review”/ OR “case report”/ OR (“systematic review” OR “case report”).ti.)

**Web-of-science**

TS=((((magnet* NEAR/2 resonance ) OR mri OR nmri OR ((mr OR nmr) NEAR/2 imag*) OR (comput* NEAR/2 tomograph*) OR ct OR cat OR radiogra* OR (x NEAR/1 ray*) OR “plain film” OR myelogra* OR (diagnos* NEAR/2 imag*) OR radiodiagnos*)) AND ((((back OR sciatic* OR lowback OR lumb* OR sacroiliac*) NEAR/5 (ache OR pain* OR aching OR complaint* OR dysfunction* OR disabilit* OR trauma* OR symptom* OR injur* OR patholog* OR problem*)) OR (fail* NEAR/2 back NEAR/2 surg* ) OR backache* OR backpain* OR schiatica OR ischia* OR lumbago OR lumboischialgia OR ((radicular OR radiculalgi*) NEAR/5 (back OR spine* OR spinal*)) OR dorsalgi*)) AND ((((spin* OR vertebra* OR intervertebr* OR disc* OR disk* OR neurologic* OR nerve*) NEAR/2 (disease* OR injur* OR tumor* OR tumour* OR neoplas* OR cancer* OR malign* OR fracture* OR hernia* OR displace* OR protru* OR avuls* OR degenerat* OR Stenos* OR Osteophytos* OR entrap* OR compress* OR inflammat* OR disorder* OR rupture* OR disrupt*)) OR Radiculopath* OR polyradiculopath* OR Spondylarthrit* OR Spondyloarthrit* OR Spondylit* OR Spondylolisthes* OR Spondylolys* OR Discitis OR Osteoporo* OR Osteoarthrit* OR Osteosclero* OR Ankylos*)) AND ((cohort* OR (follow* NEAR/1 (up OR ups)) OR followup* OR longitudinal* OR prospectiv* OR retrospectiv* OR (case NEAR/1 control*) OR historical* OR epidemiolog* OR (cross NEAR/1 section*))) NOT ((child* OR infan* OR adolescen*) NOT (adult*)) NOT ((animal* OR rat OR mouse OR rats OR mice OR murine) NOT (human* OR patient*))) AND DT=(article) NOT TI=(“systematic review” OR “case report”)

**Pubmed publisher**

(“magnetic resonance imaging”[mh] OR “Nuclear Magnetic Resonance”[mh] OR “Tomography, X-Ray Computed”[mh] OR radiography[mh] OR radiography[sh] OR “diagnostic imaging”[mh] OR radiodiagnosis[mh] OR (magnetic resonance*[tiab] OR mri OR nmri OR ((mr OR nmr) AND imag*[tiab]) OR (comput*[tiab] AND tomograph*[tiab]) OR ct OR cat OR radiogra*[tiab] OR x ray*[tiab] OR “plain film” OR myelogra*[tiab] OR (diagnos*[tiab] AND imag*[tiab]) OR radiodiagnos*[tiab])) AND (“back pain”[mh] OR sciatica[mh] OR (“Radiculopathy”[mh] AND (back[mh])) OR (((back OR sciatic*[tiab] OR lowback OR lumb*[tiab] OR sacroiliac*[tiab]) AND (ache OR pain*[tiab] OR aching OR complaint*[tiab] OR dysfunction*[tiab] OR disabilit*[tiab] OR trauma*[tiab] OR symptom*[tiab] OR injur*[tiab] OR patholog*[tiab] OR problem*[tiab])) OR (fail*[tiab] AND back AND surg*[tiab] ) OR backache*[tiab] OR backpain*[tiab] OR schiatica OR ischia*[tiab] OR lumbago OR lumboischialgia OR ((radicular OR radiculalgi*[tiab]) AND (back OR spine*[tiab] OR spinal*[tiab])) OR dorsalgi*[tiab])) AND (“Spinal Diseases”[mh] OR “Nervous System Diseases”[mh] OR Osteoporosis[mh] OR Osteoarthritis[mh] OR Osteosclerosis[mh] OR (((spine*[tiab] OR spinal*[tiab] OR vertebra*[tiab] OR intervertebr*[tiab] OR disc[tiab] OR discs[tiab] OR disk*[tiab] OR neurologic*[tiab] OR nerve*[tiab]) AND (disease*[tiab] OR injur*[tiab] OR tumor*[tiab] OR tumour*[tiab] OR neoplas*[tiab] OR cancer*[tiab] OR malign*[tiab] OR fracture*[tiab] OR hernia*[tiab] OR displace*[tiab] OR protru*[tiab] OR avuls*[tiab] OR degenerat*[tiab] OR Stenos*[tiab] OR Osteophytos*[tiab] OR entrap*[tiab] OR compress*[tiab] OR inflammat*[tiab] OR disorder*[tiab] OR rupture*[tiab] OR disrupt*[tiab])) OR Radiculopath*[tiab] OR polyradiculopath*[tiab] OR Spondylarthrit*[tiab] OR Spondyloarthrit*[tiab] OR Spondylit*[tiab] OR Spondylolisthes*[tiab] OR Spondylolys*[tiab] OR Discitis OR Osteoporo*[tiab] OR Osteoarthrit*[tiab] OR Osteosclero*[tiab] OR Ankylos*[tiab])) AND (“Epidemiologic Studies”[mh] OR “Cohort Studies”[mh] OR “Case-Control Studies”[mh] OR “cross-sectional studies”[mh] OR (cohort*[tiab] OR follow up*[tiab] OR followup*[tiab] OR longitudinal*[tiab] OR prospectiv*[tiab] OR retrospectiv*[tiab] OR case control*[tiab] OR historical*[tiab] OR epidemiolog*[tiab] OR cross section*[tiab])) NOT ((child[mh] NOT adult[mh])) NOT (animals[mh] NOT humans[mh]) NOT (Congresses[pt] OR Letter[pt] OR Notes[pt] OR Editorials[pt] OR “systematic review”[mh] OR “case report”[mh] OR (“systematic review”[ti] OR “case report”[ti])) AND publisher[sb]

**Google scholar**

mri|ct|radiography|radiographically|“diagnostic imaging”|radiodiagnosis “back|lumbar pain”|backache cohort|“follow up”|longitudinal|prospective|retrospective|“case control”|epidemiological|“cross sectional”
